# Health Care Provider Knowledge and Attitudes Regarding Adult Pneumococcal Conjugate Vaccine Recommendations — United States, September 28–October 10, 2022

**DOI:** 10.15585/mmwr.mm7236a2

**Published:** 2023-09-08

**Authors:** Rebecca Kahn, Lindsay Zielinski, Amber Gedlinske, Natoshia M. Askelson, Christine Petersen, Andrew M. Parker, Courtney A. Gidengil, Alison P. Albert, Angela J. Jiles, Megan C. Lindley, Miwako Kobayashi, Aaron M. Scherer

**Affiliations:** ^1^Division of Bacterial Diseases, National Center for Immunization and Respiratory Diseases, CDC; ^2^Epidemic Intelligence Service, CDC; ^3^Department of Internal Medicine, University of Iowa, Iowa City, Iowa; ^4^Department of Community and Behavioral Health, University of Iowa, Iowa City, Iowa; ^5^Department of Epidemiology, University of Iowa, Iowa City, Iowa; ^6^RAND Corporation, Pittsburgh, Pennsylvania; ^7^RAND Corporation, Boston, Massachusetts; ^8^Division of Infectious Diseases, Boston Children’s Hospital, Harvard Medical School, Boston, Massachusetts; ^9^Immunization Services Division, National Center for Immunization and Respiratory Diseases, CDC.

**Summary**

**What is already known about this topic?**

Despite the availability of effective vaccines, pneumococcal disease continues to be a significant cause of morbidity among U.S. adults. The Advisory Committee on Immunization Practices (ACIP) considers feasibility and acceptability of new policy options in its Evidence to Recommendations framework.

**What is added by this report?**

A survey of vaccine providers before the October 2022 ACIP meeting identified knowledge gaps and implementation challenges in existing recommendations. Respondents agreed with expanding the recommendations for 20-valent pneumococcal conjugate vaccine to adults at high risk who had received the 13-valent vaccine alone.

**What are the implications for public health practice?**

Interventions to facilitate implementation of the updated pneumococcal vaccine recommendations are needed. Available resources include the PneumoRecs VaxAdvisor mobile app and other CDC-developed tools, such as overviews of vaccination schedules and CDC’s strategic framework, Vaccinate with Confidence.

## Abstract

Despite the availability of effective vaccines against pneumococcal disease, pneumococcus is a common bacterial cause of pneumonia, causing approximately 100,000 hospitalizations among U.S. adults per year. In addition, approximately 30,000 invasive pneumococcal disease (IPD) cases and 3,000 IPD deaths occur among U.S. adults each year. Previous health care provider surveys identified gaps in provider knowledge about and understanding of the adult pneumococcal vaccine recommendations, and pneumococcal vaccine coverage remains suboptimal. To assess the feasibility and acceptability domains of the Advisory Committee on Immunization Practices (ACIP) Evidence to Recommendations (EtR) framework, a health care provider knowledge and attitudes survey was conducted during September 28–October 10, 2022, by the Healthcare and Public Perceptions of Immunizations Survey Collaborative before the October 2022 ACIP meeting. Among 751 provider respondents, two thirds agreed or strongly agreed with the policy option under consideration to expand the recommendations for the new 20-valent pneumococcal conjugate vaccine (PCV20) to adults who had only received the previously recommended 13-valent pneumococcal conjugate vaccine (PCV13). Gaps in providers’ knowledge and perceived challenges to implementing recommendations were identified and were included in ACIP’s EtR framework discussions in late October 2022 when ACIP updated the recommendations for PCV20 use in adults. Currently, use of PCV20 is recommended for certain adults who have previously received PCV13, in addition to those who have never received a pneumococcal conjugate vaccine. The survey findings indicate a need to increase provider awareness and implementation of pneumococcal vaccination recommendations and to provide tools to assist with patient-specific vaccination guidance. Resources available to address the challenges to implementing pneumococcal vaccination recommendations include the PneumoRecs VaxAdvisor mobile app and other CDC-developed tools, including summary documents and overviews of vaccination schedules and CDC’s strategic framework to increase confidence in vaccines and reduce vaccine-preventable diseases, Vaccinate with Confidence.

## Introduction

Effective vaccines against pneumococcal disease have been available in the United States for decades. Beginning in 2012, adults at high risk for pneumococcal disease or for experiencing its associated complications (i.e., adults with immunocompromising conditions and, beginning in 2014, all adults aged ≥65 years) were recommended to receive a single dose of 13-valent pneumococcal conjugate vaccine (PCV13) (Wyeth Pharmaceuticals LLC, a subsidiary of Pfizer Inc.) in series with an indication-specific number of doses of 23-valent pneumococcal polysaccharide vaccine (PPSV23) (Merck Sharp and Dohme Corp.). However, pneumococcus continued to be a common bacterial cause of pneumonia, bacteremia, and meningitis, resulting in significant morbidity and mortality in the United States. Before the COVID-19 pandemic, approximately 100,000 hospitalized pneumococcal pneumonia cases occurred among U.S. adults per year (*1*), with high all-cause mortality in older adults (*2*). In addition, there were approximately 30,000 invasive pneumococcal disease (IPD) cases (e.g., meningitis or bacteremia) and 3,000 IPD deaths annually among U.S. adults (*1*).

In October 2021, after licensure of two new pneumococcal conjugate vaccines (PCVs), 15-valent PCV (PCV15) (Merck Sharp and Dohme Corp.) and 20-valent PCV (PCV20) (Wyeth Pharmaceuticals LLC, a subsidiary of Pfizer Inc.) for use in U.S. adults, the Advisory Committee on Immunization Practices (ACIP) recommended PCV15 followed by 1 dose of PPSV23 or PCV20 alone for PCV-naïve adults aged ≥65 years or aged 19–64 years with certain health conditions or risk factors (*2*); neither PCV15 nor PCV20 was recommended for adults who had previously received PCV13.^§^ PCVs are considered more immunogenic than PPSV23, and PCV20 provides the broadest pneumococcal serotype coverage among currently available PCVs, whereas PPSV23^¶^ contains additional serotypes that are not included in PCV20** (*3*,*4*).

Previous surveys of health care providers identified gaps in their knowledge about and understanding of the adult pneumococcal vaccination recommendations (*5*,*6*). In addition, coverage with either PCV15 or PCV20 remains low, particularly among adults aged 19–64 years who are at high risk for acquisition of or complications from pneumococcal disease (Immunization Services Division, National Center for Immunization and Respiratory Diseases, CDC, unpublished data, 2023). The ACIP Evidence to Recommendations (EtR) framework, which includes seven domains, was used to guide discussions on expanding recommendations to include the new PCV20 as an option for any adult at high risk who had received PCV13, regardless of whether they had completed their previously recommended vaccination series. To assess the feasibility and acceptability domains of the EtR framework in anticipation of these updates to the pneumococcal recommendations to include PCV20 as an option for adults at high risk who received PCV13, the Healthcare and Public Perceptions of Immunizations (HaPPI) Survey Collaborative conducted a knowledge and attitudes survey of family physicians, general internists, and pharmacists^††^ during September 28–October 10, 2022. The survey aimed to assess 1) behaviors and attitudes regarding pneumococcal vaccination recommendations, 2) knowledge about pneumococcal vaccination recommendations, and 3) attitudes toward expanding recommendations for use of PCV20 among adults who had previously received PCV13.

## Methods

The HaPPI Survey Collaborative’s partnership among CDC, the University of Iowa, and the RAND Corporation uses a Qualtrics panel, a web-based survey tool, of 2 million U.S. health care providers. Eligibility criteria for this survey included spending ≥50% of practice time in outpatient primary care and administration of vaccines at the provider’s worksite. In this opt-in survey, provider attitudes toward the current pneumococcal vaccination recommendations and potential updates were assessed through open-ended responses and 5-point Likert scales, ranging from “strongly disagree” to “strongly agree.” The survey also assessed knowledge about pneumococcal vaccination recommendations via multiple choice and true or false questions. Knowledge questions were the same for all providers regardless of the types of pneumococcal vaccines that they indicated were available in their clinics. This activity was reviewed by CDC and was conducted consistent with applicable federal law and CDC policy.^§§^

## Results

Among 829 family physician, general internist, and pharmacist respondents who consented and were eligible to participate, 757 (91%) completed the survey (Table), although the number of respondents to each question varied. Among 751 respondents, 283 (38%) disagreed or strongly disagreed with the statement, “the current adult pneumococcal vaccine recommendations are easy to follow.” In the open-ended responses, 98 (13%) of the providers described the recommendations as “confusing,” and 61 (8%) felt that the recommendations included “too many choices.” Twenty-eight (4%) respondents reported that it is “hard to keep track of the recommended sequence.” Fourteen (2%) reported lack of knowledge of patients’ vaccination history as a challenge, and five (0.6%) noted challenges with supply of certain vaccines in clinics. Vaccine hesitancy among patients was reported as a reason for not recommending PCVs to patients by 63 (25%) family physicians, 56 (22%) general internists, and 40 (16%) pharmacists.

**TABLE:** Characteristics of respondents to adult pneumococcal vaccine knowledge and attitudes survey, by provider type (N = 757) — United States, September–October 2022*

Characteristic	Provider type, no. (%)		
	Family medicine (n = 255)	Internal medicine (n = 251)	Pharmacy (n = 251)
**Gender**			
Female	83 (32.5)	60 (23.9)	114 (45.4)
Male	166 (65.1)	187 (74.5)	133 (53.0)
Transgender	0 (—)	0 (—)	0 (—)
Other gender identity	6 (2.4)	4 (1.6)	4 (1.6)
**Race and ethnicity**			
Black or African American, non-Hispanic	6 (2.4)	13 (5.2)	11 (4.4)
White, non-Hispanic	176 (69.0)	140 (55.8)	185 (73.7)
Hispanic or Latino	13 (5.1)	13 (5.2)	14 (5.6)
Other	60 (23.5)	85 (33.9)	41 (16.3)
**No. of yrs in practice**			
0–5	22 (8.6)	25 (10.0)	12 (4.8)
6–10	48 (18.8)	47 (18.7)	42 (16.7)
11–15	26 (10.2)	34 (13.6)	67 (26.7)
16–20	55 (21.6)	37 (14.7)	32 (12.8)
≥21	104 (40.8)	108 (43.0)	98 (39.0)
**U.S. Census Bureau region^†^**			
Northeast	58 (22.7)	77 (30.7)	53 (21.1)
Midwest	73 (28.6)	54 (21.5)	66 (26.3)
South	70 (27.5)	76 (30.3)	95 (37.9)
West	54 (21.2)	44 (17.5)	37 (14.7)
**Metropolitan statistical area status**			
Metropolitan	230 (90.2)	233 (92.8)	226 (90.0)
Nonmetropolitan	25 (9.8)	18 (7.2)	25 (10.0)
**No. of patients treated in practice per mo**			
1–499	59 (23.1)	78 (31.1)	39 (15.5)
500–1,999	127 (49.8)	108 (43.0)	108 (43.0)
≥2,000	69 (27.1)	65 (25.9)	104 (41.4)

* Responses from pediatricians (251) were not analyzed.

^†^ https://www2.census.gov/geo/pdfs/maps-data/maps/reference/us_regdiv.pdf

**FIGURE 1:**
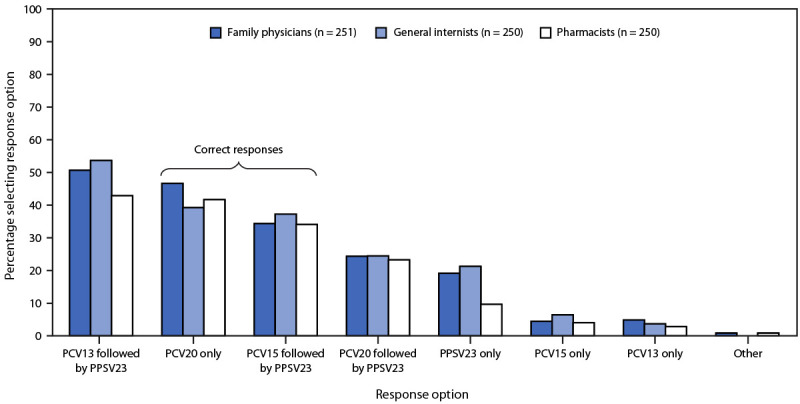
Responses* by family physicians, general internists, and pharmacists to questions about the 2021 Advisory Committee on Immunization Practices’ pneumococcal vaccine recommendations^†^ regarding which vaccines were recommended for adults aged ≥65 years who had not received any pneumococcal vaccine (N = 751) — United States, September–October 2022 **Abbreviations:** PCV13 = 13-valent pneumococcal conjugate vaccine; PCV15 = 15-valent pneumococcal conjugate vaccine; PCV20 = 20-valent pneumococcal conjugate vaccine; PPSV23 = 23-valent pneumococcal polysaccharide vaccine. * Multiple responses permitted. ^†^ https://doi.org/10.15585/mmwr.mm7104a1

Responses to the knowledge questions indicated that approximately one half of respondents were not familiar with the 2021 ACIP pneumococcal vaccination recommendations regarding PCV15 and PCV20. For example, 372 (50%) of 750 respondents incorrectly answered that adults aged ≥65 years who had previously received PCV13 should later receive PCV20. Approximately one third of 751 respondents (264, 35%) correctly answered that PCV-naïve adults aged ≥65 years should receive PCV15 followed by PPSV23, and 319 (43%) correctly selected PCV20 alone (Figure 1). CDC developed the PneumoRecs VaxAdvisor mobile app^¶¶^ to provide patient-specific pneumococcal vaccination guidance; however, among all 757 surveyed providers, 403 (53%) reported never having used the app.

**FIGURE 2:**
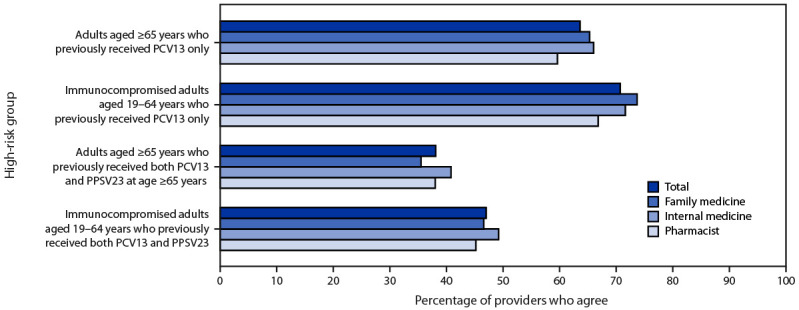
Agreement with administering 20-valent pneumococcal conjugate vaccine to different high-risk groups, by provider type (N = 751) — United States, September–October 2022 **Abbreviations:** PCV13 = 13-valent pneumococcal conjugate vaccine; PCV15 = 15-valent pneumococcal conjugate vaccine; PCV20 = 20-valent pneumococcal conjugate vaccine; PPSV23 = 23-valent pneumococcal polysaccharide vaccine.

Regarding attitudes toward expanding use of PCV20 among adults at high risk who previously received PCV13, two thirds of respondents agreed or strongly agreed with the policy option under consideration to expand the recommendations for PCV20 to adults who had received PCV13 only, including 531 (71%) who agreed with the recommendation for adults aged 19–64 years with an immunocompromising condition, and 478 (64%) who agreed with the recommendation for all adults aged ≥65 years (Figure 2). However, fewer than one half of respondents agreed or strongly agreed with the policy option under consideration to expand the recommendations for PCV20 to adults who had received both PCV13 and PPSV23, including 353 (47%) who agreed with the recommendation for adults with an immunocompromising condition and 286 (38%) who agreed with the recommendation for all adults aged ≥65 years.

## Discussion

In addition to the scientific evidence presented at the October 2022 ACIP meeting, findings from a survey among U.S. family physicians, general internists, and pharmacists conducted before the meeting were included in discussions related to ACIP’s EtR feasibility and acceptability domains (*7*). At this meeting, ACIP updated its recommendations to allow adults at high risk who received PCV13 but have not completed the recommended number of PPSV23 doses the option to receive a dose of PCV20 instead (*8*). ACIP also recommended the use of shared clinical decision-making regarding PCV20 use for adults aged ≥65 years who had completed recommended vaccination with both PCV13 and PPSV23.

The survey found that whereas providers recognize the value of pneumococcal vaccines, a substantial proportion found that the nuances of the recommendations, which varied based on patients’ vaccination history and underlying medical conditions, posed challenges to interpretation and implementation. This uncertainty might contribute to the low rates of administration of PCV15 and PCV20 among adults aged ≥65 years. As of December 2022, only 4.4% of Medicare beneficiaries aged ≥65 years had received PCV15 or PCV20; coverage was 7.1% among those aged 65–69 years, who are the least likely to have previously received PCV13 (Immunization Services Division, National Center for Immunization and Respiratory Diseases, CDC, unpublished data, 2023). Pneumococcal vaccination coverage also remains low among adults with underlying health conditions that increase their risk for pneumococcal disease and its complications, with coverage varying by race and ethnicity and geography (*9*,*10*). Limited awareness of the new vaccination recommendations and obstacles to ascertaining patients’ vaccination history might continue to hinder broader use of the new PCVs.

### Limitations

The findings in this report are subject to at least two limitations. First, a convenience sample was used, which limits generalizability and could introduce bias. However, respondents were evenly distributed among the different provider categories and geographically diverse. Second, although the survey asked if pneumococcal vaccines were offered at a provider’s clinic, the knowledge questions did not consider which pneumococcal vaccine types were offered in their clinics in the answer to these questions. Provider knowledge might have been limited to the vaccines available at their sites, and, therefore, might have skewed the knowledge responses of those whose clinics did not carry PCV20.

### Implications for Public Health Practice

Increasing vaccination coverage is critical to preventing pneumococcal disease. Gaps in knowledge and challenges to implementing recommendations for use of pneumococcal vaccines indicate a need to increase provider awareness and implementation of the most recent (October 2022) updated pneumococcal vaccination recommendations (*8*) and to provide tools to assist with patient-specific vaccination guidance. Resources available to address the challenges to implementing pneumococcal vaccination recommendations include the PneumoRecs VaxAdvisor mobile app and other CDC-developed tools, including summary documents and overviews of vaccination schedules and CDC’s strategic framework to increase confidence in vaccines and reduce vaccine-preventable diseases, Vaccinate with Confidence.*** This framework focuses on protecting communities, empowering families, and stopping misinformation from eroding public trust in vaccines. Educational interventions in the form of point-of-care decision tools, continuing medical education modules, and educational sessions at conferences might also be beneficial. Addition of more vaccines to the adult immunization schedule highlights the need for a harmonized approach to communicating new vaccine recommendations to providers from CDC and for better understanding of potential barriers to implementation of these recommendations.

## Footnotes

* These authors contributed equally to this report.

† These senior authors contributed equally to this report.

§ These adults were recommended to receive PPSV23 according to earlier recommendations. In 2012, ACIP recommended PCV13 use in series with PPSV23 for adults with an immunocompromising condition, cerebrospinal fluid leak, or cochlear implant. In 2014, all adults aged ≥65 years were recommended to receive PCV13 in series with PPSV23. In 2019, the recommendation was updated and shared clinical decision-making was recommended regarding PCV13 use for adults aged ≥65 years without an immunocompromising condition, cerebrospinal leak, or cochlear implant.

¶ Serotypes included in PPSV23: 1, 2, 3, 4, 5, 6B, 7F, 8, 9N, 9V, 10A, 11A, 12F, 14, 15B, 17F, 18C, 19F, 19A, 20, 22F, 23F, and 33F.

** Serotypes included in PCV20: 1, 3, 4, 5, 6A, 6B, 7F, 8, 9V, 10A, 11A, 12F, 14, 15B, 18C, 19A, 19F, 22F, 23F, and 33F.

†† Pediatricians were also surveyed, but their responses were not analyzed.

§§ 45 C.F.R. part 46, 21 C.F.R. part 56; 42 U.S.C. Sect. 241(d); 5 U.S.C. Sect. 552a; 44 U.S.C. Sect. 3501et seq.

¶¶
https://www.cdc.gov/vaccines/vpd/pneumo/hcp/pneumoapp.html

***
https://www.cdc.gov/vaccines/partners/vaccinate-with-confidence.html
